# ‘We stay silent and keep it in our hearts’: a qualitative study of failure of complaints mechanisms in Malawi’s health system

**DOI:** 10.1093/heapol/czad043

**Published:** 2023-11-16

**Authors:** Maryam Chilumpha, Gertrude Chatha, Eric Umar, Martin McKee, Kerry Scott, Eleanor Hutchinson, Dina Balabanova

**Affiliations:** Department of Health Systems and Policy, Kamuzu University of Health Sciences, Private Bag 360, Chichiri, Blantyre, Malawi; Department of Health Systems and Policy, Kamuzu University of Health Sciences, Private Bag 360, Chichiri, Blantyre, Malawi; Department of Health Systems and Policy, Kamuzu University of Health Sciences, Private Bag 360, Chichiri, Blantyre, Malawi; London School of Hygiene and Tropical Medicine, United Kingdom; Johns Hopkins School of Public Health, Canada; London School of Hygiene and Tropical Medicine, United Kingdom; London School of Hygiene and Tropical Medicine, United Kingdom

**Keywords:** Malawi, complaints and redress, complaints mechanism

## Abstract

A responsive health system must have mechanisms in place that ensure it is accountable to those it serves. Patients in Malawi have to overcome many barriers to obtain care. Many of these barriers reflect weak accountability. There are at least 30 mechanisms through which Malawian patients in the public sector can assert their rights, yet few function well and, as a consequence, they are underused. Our aim was to identify the various channels for complaints and why patients are reluctant to use them when they experience poor quality or inappropriate care, as well as the institutional, social and political factors that give rise to these problems. The study was set in the Blantyre district. We used qualitative methods, including ethnographic observations, focus group discussions, document analysis and interviews with stakeholders involved in complaint handling both in Blantyre and in the capital, Lilongwe. We found that complaints mechanisms and redress procedures are underutilized because of lack of trust, geographical inaccessibility and lack of visibility leading to limited awareness of their existence. Drawing on these results, we propose a series of recommendations for the way forward.

Key messages

- Despite the existence of over 30 mechanisms for submitting complaints and seeking redress in cases of mistreatment and corrupt practices leading to inadequate services in the public health system, in practice these are rarely used.
- Key factors that prevent health users from seeking redress include lack of trust of the mechanisms available, inaccessibility for the majority due to lack of visibility and distance, and lack of awareness of the options open to them. The most accessible channels at community level are also the most inactive, while those with legal powers to pursue claims are inaccessible to most people.
- The existing complaints mechanisms face constraints due to institutional, social and political factors including financial and staff shortages, poorly defined roles and lack of political stimulus to act.
- People submitting complaints need to be informed of their rights, be empowered and able to access appropriately designed, funded and independent channels that are seen as trustworthy.

## Introduction

For a health system to be responsive to community needs there must be mechanisms by which the system is accountable to those it serves. Interest in accountability of health systems has grown, especially since 1989 when the World Bank introduced the concept of ‘good governance’ to health systems (World Bank, 1989). In its 2000 *World Health Report*, the World Health Organization identified responsiveness as a core health system goal (World Health Organization, 2000; Gaventa and Barrett, 2010; Pyone *et al.*, 2017). One key aspect of responsiveness is accountability of a system to its users. The Sustainable Development Goals built upon this concept, with goal 16.6 being to ‘develop effective, accountable and transparent institutions’ (United Nations, 2016). These developments inspired the implementation of a variety of social accountability strategies. Some seek to incorporate citizen engagement and monitoring into the system, creating close-to-community structures such as health committees, public information systems and community scorecards. Others create vehicles for patients to address specific issues, such as complaints boxes or hotlines and other grievance redress systems (Thi Thu Ha *et al.*, 2015).

Effective grievance redress processes have two components: space for patients to express their complaints, and capacity to respond (Mirzoev and Kane, 2018). If either is missing, the problem is unlikely to be addressed (Hsieh, 2011b; Levin and Hopkins, 2014; Catron *et al.*, 2016) and citizens will become frustrated, disengaged from the health system, or even resort to violence (Bawaskar, 2014; McMahon *et al.*, 2014, *The Lancet*, 2014; Kar, 2017). Ultimately, there will be a loss of public confidence in the ability of the health system to respond to complaints (Standing, 2004; Vian, 2008). Effective grievance redress offers justice for those experiencing harm and reduces the risk of repetition, establishing accepted norms of transparency and accountability (Fox, 2015).

While grievance redress strategies have proliferated, there is mixed evidence of their effectiveness. If they are to work, citizens must know about them and believe that it is worth their while to engage with them because their complaints will be taken seriously and that change will result (Vian, 2013). Research in a range of settings has found low levels of awareness of mechanisms through which people can exert their rights in the health system and take action when these have been violated (Bolivar-Vargas *et al.*, 2022; Stojisavljević *et al.*, 2022). Barriers include requiring complainants to travel to distant cities, to navigate through and pay into complex bureaucratic systems, or to relive traumatic emotional experiences (Putturaj *et al.*, 2022). Some processes exclude those with limited literacy or who speak indigenous languages.

The studies cited above are part of a growing, but still limited, body of literature on citizen awareness of complaints mechanisms and the accessibility of these mechanisms, including financial barriers, pathways to accountability and procedural transparency.

This study positions itself in this space, examining the mechanisms available for seeking redress in Malawi’s health system. It is part of a larger study on corruption in the health system (such as demands for payment), so it focuses on complaints mechanisms that relate to corruption such as the Anti-Corruption Bureau. The Anti-Corruption Bureau handles complaints that relate to bribery, extortion, informal payments, pilferage and embezzlement but this mechanism does not deal with poor clinical quality of care or absenteeism. We seek to understand existing complaints channels (formal and informal), the institutions involved and how accessible and acceptable they are to the general public. We then ask what constraints and opportunities (institutional, social and political) influence whether complainants can achieve redress. Finally, we draw conclusions about what aspects of the existing complaints channels need to change if they are to work as intended, or whether new mechanisms are needed.

## Methodology

### Study setting

Malawi is a country of almost 20 million people, 84% of whom live in rural areas. About 15 local languages are spoken in the country, although the official language is English and the national vernacular is Chichewa. Only 62% of adults are literate. Health care is delivered by a mix of public, private for-profit and private not-for-profit services [provided by the Christian Health Association of Malawi (CHAM) and other faith-based organizations], with widespread scarcity of health workers, equipment and medicines. Public facilities are to provide care that is free at point of use, while nearly all private for-profit facilities charge user fees, and likewise some private not-for-profit facilities. The health system is heavily reliant on foreign aid (Adhikari *et al.*, 2019) but concerns about governance have led some donors to withdraw (Masefield *et al.*, 2020). The services that exist are overstretched; for example, while primary public health facilities should serve no more than 10 000 people, some cover up to 237 000 (Makwero, 2018). This creates obvious problems for anyone wishing to complain as there are no alternative facilities to go to for care (Jones *et al.*, 2013).

### Data collection

This study was conducted in the Blantyre district in the southern region of Malawi. Its population of 800 264 (in 2018) was served by 29 public primary health facilities and one public tertiary hospital. We chose this district because of pre-existing institutional links. We undertook participant observation, focus group discussions, in-depth interviews and desk research (Table 1).

**Table 1.** Data Collection Summary

Data collection activity	Description	N
Participant observations	Observations at the Blantyre Directorate of Health and Social Service and at three health facilities, purposively selected for wide variation by geography: one urban health centre, one peri-urban health centre, one rural health centre. Two local female researchers covered two facilities each. They were fixed at their designated facilities for the entire period. The researchers were encouraged to talk to all staff (including clinical and non-clinical members) and listen in to patient conversations. Areas and events observed: Consultation rooms Dentistry departments Vaccination clinics and queue for vaccination Antiretroviral therapy departments Waiting areas for routine consultations Management and supervisory meetings Administrative offices at the District Health Office	12 weeks (June—September 2021)
Focus group discussions	Two focus group discussions conducted in October 2021 with a group of eight. Second focus group discussion conducted in June 2022 with a group of six. Duration: 20–30 minutes. Audio recorded and transcribed. Health service users from the peri-urban health centre Health service users from the rural health centre Health service users from other health centres that are not part of the study	2 1 1 Total FGDs: 4
In-depth interviews	Conducted from April 2022—August 2022. Seven interviews done in person. Six interviews via phone. Duration: 10–20 minutes. Audio recorded and transcribed. Chair of Area Development Committee Chair of Village Health Committee Chair of Health Centre Advisory Committee Hospital Ombudsmen The secretary of an Area Development Committee A representative of a council that handles health user complaints Member of Parliament from a constituency in urban Blantyre Councillor from one of the urban wards that has one of the health facilities that have been a part of this study Two employees of two health-rights-based NGOs A representative of Blantyre District Council A representative of Blantyre City Assembly A representative of the Ministry of Health and Population	1 1 1 2 1 1 1 1 2 1 1 1 Total IDIs: 13
Policy review	Review of Malawian laws, acts and guidebooks relevant to complaints and grievance redressal in the health system. Documents collected and examined include: Malawi Government. *Chief’s Act*. 1967. Malawi Government. *Medical Practitioner and Dentist Act*. 1987. Malawi Government, 1994. Malawi Government. *Nurses and Midwives Act*. 1995a. Malawi Government. *Corrupt Practices Act*. 1995b. Malawi Government, 1998. Malawi Government. *Public Audit Act*. 2003 Malawi Government. *Police Act*. 2010. Malawi Government. *Pharmacy and Medicines Regulatory Authority Act*. 2019 Ministry of Local Government and Rural Development, 2013	Total documents: 10

Two social scientists with knowledge of health system governance and public administration collected the data (MC and GC). Both were trained in participant observation and in writing and analysing fieldnotes. Data collection in the health facilities began with two weeks of general observations followed by 10 weeks of research focused on a specific topic, one of which was the complaints and redress process. Permission was acquired from the Blantyre Directorate of Health and Social Services (DHSS) district office, which manages all public primary health-care facilities in the district. Officers in charge or their representatives were the first to be approached at the start of the study. Visits were made during mornings, afternoons, evenings and occasionally at the weekends. At the DHSS, the researcher conducting observations moved between offices, assisted some of the administrative staff with tasks, and held informal discussions with officials. Gradually, the district health officials agreed that the researcher could also attend their meetings.

Fieldnotes were written contemporaneously, and emerging findings were discussed each week by the researchers, the Malawian PI (MC, GC and EU), anthropologist (EH), and health systems researcher (DB). Where appropriate, the findings were fed into adjustments to the research plan, particularly the schedule of observations and sampling of respondents.

The focus group discussions covered the following domains: recent experiences with health-care-seeking; relationships between patients and health-care providers; options available if community members felt they had been mistreated at a government health facility; and reasons for not reporting grievances. In-depth interviews covered the following domains: the accessibility of the respondent’s organization to the public; number of complaints received per month; constraints on receiving and handling complaints; the process of redress (Table 2).

**Table 2.** In-depth interviews

Participant	Number of participants	Mode
A representative of a council that handles health user complaints	1	On the phone
A representative of Blantyre City Assembly	1	In person
A representative of Blantyre District Council	1	In person
A representative of the Ministry of Health and Population	1	On the phone
Chairperson of Area Development Committee	1	In person
Chairperson of Health Centre Advisory Committee	1	In person
Chairperson of Village Health Committee	1	In person
Councillor (from one of the urban wards that has one of the health facilities that have been a part of this study)	1	On the phone
Employees health of two different rights-based NGOs	2	On the phone
Hospital Ombudsman	2	In person and on the phone
Member of Parliament from a constituency in urban Blantyre	1	On the phone
The secretary of an Area Development Committee	1	On the phone

### Analysis

Fieldnotes were read and reviewed by the two researchers and the multidisciplinary team while the fieldwork was being undertaken. Key themes related to the research questions were identified at weekly team discussions informing further data collection. Once the fieldwork was finalized, the fieldnotes and transcripts were uploaded into NVivo data analysis software and coded by each fieldworker using a mix of deductive and inductive coding. First, emerging themes (both dominant and divergent) were coded inductively by each researcher and were then compared and discussed with the broader team on a weekly basis. This led to the development of a coding framework. Following that, data were re-examined against a priori interests such as institutional barriers and enablers of complaints processes in low-resource settings, thus enriching the coding framework. The analytical approach was therefore broadly consistent with the framework approach (Pope *et al.*, 2000), but it was applied in a flexible manner, to capture counterintuitive findings.

### Ethical approval

Formal consent was obtained from the authors’ institutes in Malawi and the UK. Participants in in-depth interviews and focus group discussions gave informed formal consent. We developed a comprehensive ethics procedure for the ethnography. We held a formal meeting with staff at each facility to present the project, provide information sheets and respond to their questions. We sought written consent from officers in charge of facilities to undertake research at their facilities. Following this, we sought verbal consent from research participants, constantly monitoring any emerging sensitivities and possible distress to those observed.

## Results

We begin with a summary of common grievances reported during focus group discussions and identified during ethnographic observations. We then map the many options for grievance redress in Malawi, explore reasons why they are rarely used, and ask why those that are lodged are rarely resolved.

### Common grievances reported by health-care users

Patients reported many challenges when seeking care. Many grievances reflect a shortage of resources but there were examples of misconduct by health workers. These included reports of sexual harassment of female patients, harsh treatment of women in labour, and rushed and inconsiderate treatment of both male and female patients. Patients reported being given a diagnosis before they were able to explain their symptoms, receiving insufficient medication, and having one health issue addressed while other symptoms were ignored.

### Mechanisms to manage complaints in the Malawian health system and their characteristics

We identified over 30 channels through which citizens should be able to make complaints about health workers (Table 3, with additional detail in supplementary Table S1).

**Table 3.** Potential grievance redress mechanisms for health-care issues in Malawi at the community, health facility, district and national levels

Mechanisms	Community	Health facility	District	National
*Advocacy-based mechanisms:* Can refer complaints to other bodies and advocate for redressal	Traditional leader [informal] Religious leaders [informal] Village Health Committees [informal] Ward counsellor [semi-formal] Area/Community Development Committee (ADC/CDC) [semi-formal] Members of Parliament [semi-formal]	Suggestion box [formal]		NGO/Rights-based organizations [formal] Quality Management Directorate [semi-formal] National Local Government Finance Committee [formal] Office of the Auditor General [formal] Drug Theft Investigation Unit [formal]
Mediating mechanisms: can mediate disagreements	Traditional/local court [informal]			
Employment-based mechanisms: can suspend, transfer or fire health workers		Health Centre Advisory Committees [formal] Health Facility Officer in Charge [semi-formal] Hospital Ombudsman (within health facilities) [formal]	District Council/District Commissioner [formal] City Council [formal] Directorate of Health and Social Services (District Health Office) [formal] District Hospital Ombudsman [formal]	Minister of Health and Population [informal] Minister of Local Government and Rural Development [semi-formal] Ministry of Health and Population [informal] Ministry of Local Government and Rural Development [formal] Anti-Corruption Bureau [formal] Office of the Ombudsman [formal] Medical Council of Malawi [formal] Nurses and Midwives Council of Malawi [formal] Pharmacy and Medicines Regulatory Authority [formal]
Legal mechanisms: can charge or convict health workers			Malawi police services (district) [formal] Judiciary (Lower Grade 1, 2, 3, 4 Magistrate Courts and Local Courts) [formal]	Malawi police services (national) [formal] Judiciary (Supreme Court of Appeal, High Court, Chief Resident Magistrates; Principal Resident Magistrates; Senior Resident Magistrates; Resident Magistrates) [formal] Parliamentary Committee on Health [formal]

These mechanisms cluster around certain roles (advocacy, mediation, employment and legal power) and exist at different levels of the health system (community, health facility, district or national). All can accept complaints but not all can process and resolve them. The main types of mechanism are described below.

#### Roles: from advocacy to legal mechanisms

Mechanisms relying on the power of advocacy cannot resolve complaints. Instead, they can accept complaints (often serving as the front line), refer complaints to other authorities and advocate for change by health facilities, health-care providers, or elsewhere. As depicted in Table 1, almost every mechanism accessible at the community level falls within this domain. The only exception is the traditional court, which also provides a forum for mediation where complaints can be investigated and each party can present their case, with resolution achieved through socially agreed mediation rules. While the traditional court can award compensation to victims, in money or livestock, its rulings are not legally binding although they do have moral authority.

Patients saw advocacy mechanisms, and particularly those involving traditional and religious leaders, as well as Village Health Committees, as easier to access due to their proximity. People involved in these structures are often neighbours or members of the same social networks as those seeking redress. A patient who was afraid of reporting their grievance to the health facility personally could ask someone in these bodies to lodge a complaint with the hospital ombudsman on their behalf. If such a complaint is not addressed these bodies can advocate on behalf of the complainant.

Mechanisms rooted in employment structures and procedures make it possible to discipline health workers who breach the terms of their contract or provide inadequate clinical care. Sanctions involve issuing warnings, suspensions, transfers and revoking the licence of health practitioners. Those working within the health system reported that most grievances come to notice via the health facility hospital ombudsman. If they cannot handle the complaint, then they should do so in conjunction with the Health Centre Advisory Committee’s (HCAC) sub-committee on complaints.

Finally, complainants can use the legal system to press charges against health workers so that a formal legal judgement is obtained to convict or sanction health system actors who violate patient rights. These mechanisms are concentrated in the four cities of Malawi, with a few at the district level.

Importantly, different types of channels are not accessed in sequence: those making complaints do not progress from community to higher levels of the health system or the courts or through the public administration system. Instead, those making complaints must select an entry point based on their (often limited) understanding of the options and personal ability to navigate the system. As a result, an individual’s choice of which organization to complain to generally reflects their familiarity with it, their socio-economic status, their social networks, and the ease with which they can access the organization.

### Reasons for the low utilization of existing complaints mechanisms

#### Lack of awareness

Despite the frequently expressed grievances and the existence of over 30 redress mechanisms in the Malawi health system, none of the patients we spoke to had reported their experience or sought redress in any way. Many were not aware of any grievance redress options.

*Yes, we might be afraid because we just don’t know where to go, who to meet. So, it is not fear, but we just don‘t know. At least if they would tell us that if you encounter any problems this is where you should go and meet this person. As we’ve mentioned we don‘t know who the ombudsman is. They have never told us in which office the [hospital] ombudsman is found. We don’t know where to go or who we can find. (FGD 02, female health system user, 2021).*

*People outside, the chiefs etc. don’t know that this health centre has a committee or the patron they don’t know them. People are ignorant of this. So even if a person has encountered a problem, the patron does not come forward so that the people may know them. (FGD 02, male health systems user, 2021)*

#### Lack of access to most mechanisms

Even if a patient knew who to complain to, it was not always possible to gain access to the individuals occupying those offices. Those at the community level who were most accessible often lacked the power to resolve grievances. In contrast, members of parliament (MPs) could advocate successfully for their constituents but often lived elsewhere and/or spent considerable time away on official duties.

As noted, patients were encouraged to approach the hospital ombudsmen or HCAC but often they did not know who these individuals were and, even if they did, the individuals concerned were frequently absent from health facilities. A hospital ombudsman described how s/he rarely had time to perform community outreach and lacked a dedicated office that is accessible and appropriate, in terms of privacy, for those making a complaint:

*It is hard to find me at the health centre because I do not have a proper office and as such I am usually based at the DHO [Directorate of Health and Social Services Office]. At the same time many complainants do not want to be seen interacting with me as they prefer anonymity as they fear they will not be assisted accordingly. I am also supposed to give daily health talks as a means of making the patients aware of how they can reach me as hospital ombudsman since different patients come every day. It is not possible as I am usually busy with my HSA (health surveillance assistant) duties. This makes it difficult for health users to know about me as a hospital ombudsman and how they can find me. (Hospital ombudsman, 2022).*

Any patients seeking to complain directly to officers at the DHSS face another set of access barriers. Absenteeism is commonplace at the DHSS and the officers who handled complaints were often only available on a Monday, spending the rest of the time in the field.

Most of the legal mechanisms through which grievances can be reported require the complainant to visit the cities of Lilongwe, Blantyre or Mzuzu. To place a complaint with the Medical Council of Malawi and the Nurses and Midwives Council of Malawi, for instance, people need to either attend in person, call them via their toll-free number, write an email or send a letter. Since these mechanisms are only available in three places it is often difficult for those who live far away to access them. At the same time their toll-free line is not well known as the number is not available on their websites and the internet is not accessible to the majority of the population. As a result, these councils have a very low rate of complaints (an average of two a month) (Representative of Council for Health User Complaints, 2022).

The only channel that is widely known to the public is the Anti-Corruption Bureau, a national structure with headquarters in Lilongwe and offices in Blantyre, Zomba and Mzuzu cities. It also works closely with various public sector organizations. Many government offices display a poster about the Anti-Corruption Bureau and how to reach them. However, lodging a complaint with this Bureau is only possible in writing. Although suggestion boxes are now provided in public health facilities, these are only useable by those who can read and write at a level sufficient to explain their often-complex stories.

#### Fear of reprisal and a lack of trust in the mechanisms

Some patients who were aware of existing grievance mechanisms and were able to overcome access barriers still failed to engage due to fear and lack of trust. In particular, they feared reprisal from health workers and did not trust the complaint resolution processes to protect them.

*We encounter many problems, and we know where to take our grievance, but we are afraid of taking the matter forward. They will ask you how do you know that this is what happens? Who told you? As such because we are afraid of being asked these questions, we stay silent and keep it in our hearts even though we face many problems and we have grievances. (FGD 02, male health system user, 2021)*

The ombudsmen were not considered sufficiently independent to be trusted with confidential complaints because they work within the health facilities and are health-care providers themselves. There is an expectation that providers protect each other in cases of complaints regardless of their public roles.

*The problem that we have is where we can go and lodge our grievances, it is there at the same health facility and we are complaining to the same people. So we don’t see how these people would help us and they can change (FGD 04, male health systems user, 2022)*

Fear was a major deterrent to making a complaint, a point reiterated by several health providers and a key informant from the Quality Management Directorate. Even after lodging initial complaints, patients rarely appeared at formal hearings because of concern that their problems would not be addressed fairly and that they would face retaliation the next time they and their families needed care.

*It is a closed system; complainants fear they will not receive medical attention the next time they visit that health facility. (A representative of the Ministry of Health and Population, 2022).*

Apart from fear of reprisals, patients and informants from NGOs doubted the general effectiveness of grievance redress processes. These respondents questioned whether hospital ombudsmen have the actual power to discipline their colleagues or would be willing to do so, particularly if the complaint is against a senior clinician.

*Being that a hospital ombudsman is a junior officer, when a complaint is against someone more senior to them such as an officer in charge or a head of department it becomes a challenge for them to resolve a matter. (NGO representative, 2022).*

It was suspected that suggestion boxes were tampered with or stolen by health providers (Hospital ombudsman, 2022). This is especially the case with complaints related to corrupt behaviour and mistreatment, given the potential impact of sanctions on the providers’ reputation.

*If there is a suggestion box it’s doubtful because the ones that are going to take the suggestion box are the same people that do not want to assist you. So, we don’t know what we can do. (FGD 04, male health systems user, 2022)*

### Constraints to the effective redress of complaints

Despite the existence of a wide range of mechanisms for redress of complaints, respondents believed that genuine accountability was rare, often because of limited resources to fully investigate and act on the complaints as well as interference by politically powerful individuals.

#### Lack of funding, resources and incentives to deal with redress

At the community level, Area Development Committees were limited in the extent to which they could advocate due to their almost complete lack of government funding. Members are volunteers who rely on allowances in order to attend meetings and other functions.

At the health facility level, the hospital ombudsmen reported receiving insufficient funding to share information and engage with the public (e.g. using posters), to buy credit for their mobile phones to call complainants when additional information was needed, or to obtain the stationery needed to keep proper records and provide progress reports. HCACs are expected to meet once a month but, given their scarce funding, are not always able to do so, delaying complaint resolution. One HCAC representative described how the lack of resources curtails their ability to perform their duties and that the lack of financial benefits meant that incentives to tackle health users’ complaints were limited.

#### Interference by social and political networks

The ethnographic observations revealed how there is often a struggle to investigate complaints because officers-in-charge are rarely forthcoming with evidence against their colleagues. Typically, they will claim that there is a lack of evidence of the health provider engaging in any wrongdoing that would require disciplinary action.

Participants in focus group discussions described their suspicion that the ADC and the HCAC were more concerned with their own political agenda than with resolving issues within the local community. For example,

*…It is true here at the health facility that there is a lot of politics. Any party that wins, if there is a committee, they try their best to put their people at this health centre so that they can collude with these people to commit corruption and other things without any difficulty. This is what has been happening. […] You will discover that the committee is full of DPP (Democratic Progressive Party). The ones that have just come (the current MPC government) are probably busy trying to remove them even though some of them their term is not over. Such things make a poor person to continue to suffer because of certain politicians. (FGD 02, male health systems user, 2021)*

Likewise, those who were able to take a complaint against the health system to their local MP could find them unwilling to help. Fear of a political backlash means that complainants become disillusioned and lose the will to seek redress (Member of Parliament, 2022).

*We fear being accused of political interference when we attempt to address these complaints with government health facilities. They would go to the media and spin it as politicians interfering in departmental business. This is a huge barrier in tackling the corruption which happens openly such as asking patients for informal payments. (Member of Parliament, 2022).*

## Discussion

We examined the different mechanisms through which patients can raise complaints and seek redress in the Malawian health system, exploring how they function in practice. Despite a commitment by the government and others to address corruption and mistreatment of service users and to establish viable mechanisms for redress that are accessible to the population, there is little understanding of what is happening in reality. While our findings are of immediate relevance to Malawi, they may also be relevant to other countries in sub-Saharan Africa.

While we identified over 30 potential grievance redress mechanisms, these were underutilized. Few people knew that they could access these mechanisms when they had complaints related to rent-seeking in the health sector. Those who were familiar with the mechanisms did not trust them to respond to and resolve their complaints. The most powerful mechanisms are those that make it possible to sanction health-care workers or press criminal charges but they are the least accessible. The mechanisms that are most accessible at community level are the weakest. All of the bodies tasked with redressing grievances lacked robust procedures for handling complaints and were severely underfunded. They often had few incentives to provide redress and were subject to the power of social and political networks and alliances.

Our study has certain limitations, in particular the challenge of engaging with systems for dealing with complaints at a national level. This raises an important question for the academic community, which has often struggled to tackle areas that are by their nature opaque, whereas others, in particular investigative journalists, have been more successful. The Covid-19 pandemic reduced the amount of time that the researchers were able to spend in the field. However, this was compensated for through follow-up interviews with particularly knowledgeable informants.

Our findings echo other research from Malawi revealing the limited effectiveness of complaints channels at community level and the inaccessibility of those at higher levels. The Malawian Quality Management Directorate, Office of the Ombudsman and WISH found that although many patients made complaints, few used the mechanisms that were at their disposal (Quality Management Directorate, Office of the Ombudsman and WISH, 2021). Jones *et al.* (2013) reported that local structures, such as Malawi’s Village Health Committees, were the easiest for patients to access but they were not recognized as effective. Area Development Committees are limited by members’ lack of formal education, and access to information and necessary resources, and the HCACs rarely receive training on how to execute their roles (Jones *et al.*, 2013).

In the past, traditional courts had a legal mandate to adjudicate complaints in the health sector. However, they were stripped of their power in 1994 following their actions in punishing political opponents of the regime led by Hastings Banda (the first post-independence president) (Ubink, 2016). In 2011, a Parliamentary bill allowed traditional courts to adjudicate under customary law but this arrangement has not yet been implemented (Ubink and Weeks, 2017). Another constraint to redressing complaints is the lack of designated funding for HCACs, which delays the resolution of complaints. Additionally, lack of resources constrains them from performing their duties to the fullest capacity as they lack financial motivations such as a salary or allowances to incentivize them to deal with complaints that are potentially time-consuming and sensitive (Jones *et al.*, 2013).

Gloppen and Kanyongolo argue that most complainants are unaware that it is their right to take their complaint to court and that magistrate courts are not inaccessible to many Malawians (Gloppen and Kanyongolo, 2007). At the district and national levels, most complainants do not live close to a magistrate court; travel and legal representation are costly and the number of lawyers in Malawi is low and very few take on pro bono cases (Gloppen and Kanyongolo, 2007). As Kalembera argues, in rural areas most magistrate courts that are accessible are Third Grade Magistrate Courts whose jurisdiction is to try cases that have a sentence of 3 years or less (Kalembera, 2016).

Additionally, limited funding, staff and resources at the Office of the Ombudsman have a bearing on a heavy backlog of cases (Hussein, 2005). National institutions such as the Anti-Corruption Bureau are described as lacking in expertise and skills to carry out investigations when they receive a complaint (Hussein, 2005). The implications of this are delays in commencing criminal proceedings (Kamanga, 2008).

Importantly, there is increasing literature that demonstrates the influence of power, social and political networks and alliances on institutional processes that seek to improve governance. Most complaints received by the Medical Council of Malawi have been from urban university graduates in the major cities of Lilongwe and Blantyre (Ndovie, 2012). Members of Parliament and Ward councillors have a difficult working relationship, often regarding one another as political opponents (Chinsinga and Dzimadzi, 2001; Member of Parliament, 2022). Unless those grievances align with their financial and political interests, complaints are rarely regarded as serious or acted upon.

Institutions such as the Anti-Corruption Bureau also face political interference in their work (Doig *et al.*, 2006; Kamanga, 2008). Often, where a crime involves a lower ranking public figure prosecution is possible, but where the accused is of a high political ranking there appear to be very few prosecutions. Institutions that are mandated to manage and respond to complaints suffer multiple capacity constraints.

These problems found in Malawi can also be seen in other settings. Complaints mechanisms for health-care system users are often promoted by donors who push for their own interests instead of for community needs (Jones *et al.*, 2013) and so attention is frequently on safeguarding external funds for medicines and supplies. Complaints mechanisms are often standalone, only operational for the duration of particular donor-funded vertical programmes, with insufficient knowledge transfer and poor adaptation to the socio-economic and cultural contexts (Birdsall, 2004; Koch and Weingart, 2016; Niyonkuru, 2016). Complaints mechanisms in many settings continue to ignore low literacy levels among the population and the imbalance of power between the players involved (Mirzoev and Kane, 2018). A study conducted in Vietnam similarly revealed that complaints mechanisms exist in the health system but these are underused (Thi Thu Ha *et al.*, 2015). Evidence from Bangladesh also indicates that multiple parallel health user complaints systems overlap and thus create confusion for those who seek to use them (Huque *et al.*, 2021; Mirzoev *et al.*, 2021).

## Conclusion and recommendations

This paper identified over 30 potential formal and informal complaints mechanisms that exist at community, health facility, district and national levels that, in theory, enable health users to lodge complaints in a quest for redress. However, many of these mechanisms are underutilized, in part because of lack of trust (that their grievances will be addressed) but also due to inaccessibility (due to distance, poverty and sometimes no one knows how to locate them), reflecting a lack of visibility (they are not always known to the public), the distance between the complainant and where the complaint can be lodged, and a lack of awareness.

Our recommendations are, of necessity, influenced by the economic reality facing the health system in Malawi, the existing capacity as well as the pervasive lack of trust in formal systems. In order to address the problems faced by users seeking redress and to improve the existing mechanisms in Malawi, our recommendations are as follows:

1. The majority of the informal grievance channels are available at community level and already handle non-health-related complaints—enabling the population to seek redress. To minimize barriers to accessibility for poor and rural communities, these mechanisms should be strengthened and formalized, and receive better funding and effective oversight.
2. Instead of multiple and insufficiently distinguished complaints mechanisms, these should be organized in a hierarchy—within health systems and beyond. These mechanisms need to be clearly signposted to help complainants access and navigate the redress system. Efforts should seek to reduce fragmentation and improve coherence of the way these institutions act to promote resolution including an explicit process for referral or escalating complaints to more appropriate channels. The interests and capacities of the different institutions to handle complaints effectively need to be examined.

For example, this could be done by linking all the mechanisms under the Ministry of Health and Population in the Quality Management Directorate, which is already responsible for improving standards of service delivery in public health facilities and for the training of hospital ombudsmen. Furthermore, all community level mechanisms have a link to health facilities, and complaints could be formally mediated by trusted community structures rather than by relying on health systems channels. This would remove accessibility barriers and would shorten the length of time it takes for a complaint to be redressed. Grievances that cannot be resolved via the community mechanisms can be escalated to the hospital ombudsman and then to the district hospital ombudsman as is already current practice.

3. The communication approaches currently employed to empower health users on their right to complain when encountering problems at a health facility should be critically reviewed. The question is why these approaches have not been as effective in reaching the targeted audiences and empowering a larger group of health users.

Communication channels need to take into account the underlying power differentials that prevent many users from trusting and accessing formal complaints mechanisms; these mechanisms may need to be reached using informal and traditional networks, which may be seen as more supportive. Messages on how to complain and seek redress could be incorporated into routine health communication campaigns to ensure that they are familiar to health-care users.

4. Trust in those who have been tasked with complaints redressal is critical. Therefore, measures should be put in place to create processes that assure patients that their complaints will be handled fairly and that they will not face repercussions the next time they require medical attention. For example, complaint mechanisms should be made available in a way that does not jeopardize the complainant’s access to their local facilities where they can be identified as a complainant.

While these recommendations have been made to fit the Malawian context, lessons can be drawn for other settings facing similar challenges. First, there is a need to develop systems that allow complaints to be dealt with as close to the community as possible and to ensure that these systems are linked to trusted local formal and informal governance structures, while enabling hierarchical escalation when necessary. Second, lines of accountability should be clear and well understood by end users. This may be difficult where there are multiple funding pathways, some of which are controlled by donors, each with their own reporting mechanisms. Third, it is crucial to take account of power differentials and ensure that those who complain are not at risk. A deeper understanding of the institutions that are meant to respond to the complaints as well as their incentives is needed in order to understand where to intervene.

## Supplementary Material

czad043_Supp

## Reflexivity statement

The authors are a multidisciplinary team with expertise in public/global health, anthropology, psychology and communication from both HIC (United Kingdom and Canada) and LMIC (Malawi). The group consists of early, mid and senior researchers who contributed to data analysis and finalization of the article with their unique perspectives. Authors have engaged weekly and worked together consistently for the duration of study conceptualization, fieldwork, and analysis enabling them to collaboratively derive and interpret key findings.

## References

[R1] Adhikari R, Sharma JR, Smith P et al. 2019. Foreign aid, Cashgate and trusting relationships amongst stakeholders: key factors contributing to (mal) functioning of the Malawian health system. *Health Policy and Planning* 34: 197–206. 31005983 10.1093/heapol/czz021

[R2] Bawaskar HS . 2014. Violence against doctors in India. *The Lancet* 384: 955–6. 10.1016/S0140-6736(14)61629-9 25220972

[R3] Birdsall N . 2004. Seven deadly sins: reflections on donor failings. *Reform and Growth: Evaluating the World Bank Experience* 13: 181.

[R4] Bolivar-Vargas M, Alfonso-Sierra E, Bonilla J et al. 2022. How can community participation strengthen a health insurance system? The case of health insurer’s user associations in Colombia. *BMJ Global Health* 7: e009571. 10.1136/bmjgh-2022-009571 PMC9486220 36379588

[R5] Catron TF, Guillamondegui OD, Karrass J et al. 2016. Patient complaints and adverse surgical outcomes. *American Journal of Medical Quality* 31: 415–22. 25916627 10.1177/1062860615584158

[R6] Chinsinga B, Dzimadzi C. 2001. Final report: impact assessment study on the process of decentralization in Malawi. Lilongwe: Decentralization Secretariat.

[R7] Doig A, Watt D, Williams R. 2006. Hands‐on or hands‐off? Anti‐corruption agencies in action, donor expectations, and a good enough reality. *Public Administration and Development: The International Journal of Management Research and Practice* 26: 163–72.

[R8] Fox JA . 2015. Social accountability: what does the evidence really say? *World Development* 72: 346–61.

[R9] Gaventa J, Barrett G. 2010. So what difference does it make? Mapping the outcomes of citizen engagement. *IDS Working Papers* 347: 1–72.

[R10] Gloppen S, Kanyongolo FE. 2007. Courts and the poor in Malawi: economic marginalization, vulnerability, and the law. *International Journal of Constitutional Law* 1: 258–93.

[R11] Hsieh SY . 2011a. Healthcare complaints handling systems: a comparison between Britain, Australia and Taiwan. *Health Services Management Research* 91: 95–24. 10.1258/hsmr.2011.011003 21471579

[R12] Hsieh SY . 2011b. A system for using patient complaints as a trigger to improve quality. *Quality Management in Healthcare* 20: 343–55. 10.1097/QMH.0b013e318222e73b 21971031

[R13] Huque R et al. 2021. Patient feedback systems at the primary level of health care centres in Bangladesh: a mixed methods study. *Sage Open* 1: 15.

[R14] Hussein M . 2005. Combating corruption in Malawi: an assessment of the enforcing mechanisms. *African Security Studies* 14: 91–101.

[R15] Jones S et al. 2013. Local perceptions, participation and accountability in Malawi’s health sector. *Norad Report 2/2013*. Oxford Policy Management, May 2013. https://www.oecd.org/derec/norway/NORWAY_LocalPerceptiosParticipationAccountabilityinMalawisHealthSector.pdf, accessed 2 May 2023.

[R16] Kalembera SA . Jurisdictional limits for magistrates are hindering access to justice in Malawi. Goal 16 of the Sustainable Development Goals: Perspectives from Judges and Lawyers in Southern Africa on Promoting Rule of Law and Equal Access to Justice 2016. 103–11. https://www.southernafricalitigationcentre.org/wp-content/uploads/2017/08/GOAL-16-Book.pdf, accessed 2 May 2023.

[R17] Kamanga I . 2008. Combating corruption: challenges in the Malawi legal system. *UNAFEI Resource Material Series* 76: 153–19.

[R18] Kar SP . 2017. Addressing underlying causes of violence against doctors in India. *The Lancet* 389: 1979–80. 10.1016/S0140-6736(17)31297-7 28534749

[R19] Koch S, Weingart P. 2016. *The delusion of knowledge transfer: the impact of foreign aid experts on policy-making in South Africa and Tanzania*. Cape Town, South Africa: African Minds.

[R20] The Lancet . 2014. Violence against doctors. Why China? Why now? What next? *The Lancet* 383: 1013. 10.1016/S0140-6736(14)60501-8 24656183

[R21] Levin CM, Hopkins J. 2014. Creating a patient complaint capture and resolution process to incorporate best practices for patient-centered representation. *Joint Commission Journal on Quality and Patient Safety/Joint Commission Journal* 40: 484–92. 10.1016/s1553-7250(14)40063-1 26111366

[R22] Makwero MT . 2018. Delivery of primary health care in Malawi. *African Journal of Primary Health Care and Family Medicine* 10: 1799. 10.4102/phcfm.v10i1.1799 PMC6018651 29943590

[R23] Malawi Government . 1967. Chief’s Act. https://www.malawilii.org/akn/mw/act/1967/39/eng%402014-12-31, accessed 2 May 2023a.

[R24] Malawi Government . 1987. Medical Practitioner and Dentist Act. https://media.malawilii.org/files/legislation/akn-mw-act-1987-17-eng-2014-12-31.pdf, accessed 2 May 2023b.

[R25] Malawi Government . 1994. Constitution of Malawi. https://www.constituteproject.org/constitution/Malawi_2017.pdf?lang=en, accessed 2 May 2023.

[R26] Malawi Government . 1995a. Nurses and Midwives Act. https://media.malawilii.org/files/legislation/akn-mw-act-1995-16-eng-2014-12-31.pdf, accessed 2 May 2023.

[R27] Malawi Government . 1995b. Corrupt Practices Act. https://www.malawilii.org/akn/mw/act/1995/18/eng%402014-12-31, accessed 2 May 2023.

[R28] Malawi Government . 1998. Local Government Act. http://www.ndr.mw:8080/xmlui/bitstream/handle/123456789/1156/Local%20Government%20Act%2C%201998.pdf?sequence=1, accessed 2 May 2023.

[R29] Malawi Government . 2003. Public Audit Act. https://www.malawilii.org/akn/mw/act/2003/6/eng%402014-12-31, accessed 2 May 2023c.

[R30] Malawi Government . 2010. Police Act. https://www.malawilii.org/akn/mw/act/2010/12/eng%402014-12-31, accessed 2 May 2023d.

[R31] Malawi Government . 2019. Pharmacy and Medicines Regulatory Authority Act. https://www.malawilaws.com/Malawipdf2019/Act%209%20of%202019.pdf, accessed 2 May 2023.

[R32] Masefield SC, Msosa A, Grugel J. 2020. Challenges to effective governance in a low-income healthcare system: a qualitative study of stakeholder perceptions in Malawi. *BMC Health Services Research* 20: 1–6. 10.1186/s12913-020-06002-x PMC7734892 33317520

[R33] McMahon SA, George AS, Chebet JJ et al. 2014. Experiences of and responses to disrespectful maternity care and abuse during childbirth; a qualitative study with women and men in Morogoro Region, Tanzania. *BMC Pregnancy and Childbirth* 14: 268. 10.1186/1471-2393-14-268 PMC4261577 25112432

[R34] Ministry of Local Government and Rural Development . 2013. Guidebook on the Local Government System in Malawi. https://www.resakss.org/sites/default/files/Malawi%20MLGRD%202013%20Guidebook%20on%20the%20Local%20Government%20System%20in%20Malawi.pdf, accessed 2 May 2023.

[R35] Mirzoev T, Kane S. 2018. Key strategies to improve systems for managing patient complaints within health facilities—what can we learn from the existing literature? *Global Health Action* 11: 1458938. 10.1080/16549716.2018.1458938 PMC5912438 29658393

[R36] Mirzoev T, Kane S, Al Azdi Z et al. 2021. How do patient feedback systems work in low-income and middle-income countries? Insights from a realist evaluation in Bangladesh. *BMJ Global Health* 6: e004357. 10.1136/bmjgh-2020-004357 PMC7878124 33568396

[R37] Ndovie RM . 2012. Content analysis of patients/guardians’ complaints against medical practitioners and health facilities lodged at Medical Council of Malawi from January 2007 to December 2011. *Dissertation*. Blantyre: College of Medicine, Kamuzu University of Health Sciences.

[R38] Niyonkuru F . 2016. Failure of foreign aid in developing countries: a quest for alternatives. *Business and Economics Journal* 7: 1–9.

[R39] Pope C, Ziebland S, Mays N. 2000. Analysing qualitative data. *British Medical Journal* 320: 114–6. 10625273 10.1136/bmj.320.7227.114 PMC1117368

[R40] Putturaj M, Krumeich A, Nuggehalli Srinivas P et al. 2022. Crying baby gets the milk? The governmentality of grievance redressal for patient rights violations in Karnataka, India. *BMJ Global Health* 7: e008626. 10.1136/bmjgh-2022-008626 PMC9150157 35623644

[R41] Pyone T, Smith H, van den Broek N. 2017. Frameworks to assess health systems governance: a systematic review. *Health Policy and Planning* 1: 710–22. 10.1093/heapol/czx007 PMC5406767 28334991

[R42] Quality Management Directorate, Office of the Ombudsman and WISH . 2021.The rapid assessment of the Hospital Ombudsman functionality and client satisfaction survey in Kasungu and Lilongwe Districts. Lilongwe: Malawi Government.

[R43] Standing H . 2004. Understanding the ‘demand side’ in service delivery. Definitions, frameworks and tools from the health sector. Issues Paper Private Sector, DFID Health Systems Resource Centre. https://www.heart-resources.org/wp-content/uploads/2012/10/Understanding-the-demand-side-in-service-delivery.pdf, accessed 7 August 2023.

[R44] Stojisavljević S, Đikanović B, Vončina L et al. 2022. The challenge of ensuring elderly people can access their health insurance entitlements: a mixed methods study on the Republic of Srpska’s Protector of Patients’ Health Insurance Entitlements. *BMJ Global Health* 7: e009373. 10.1136/bmjgh-2022-009373 PMC9476123 36109016

[R45] Thi Thu Ha B, Mirzoev T, Morgan R. 2015. Patient complaints in healthcare services in Vietnam’s health system. *SAGE Open Medicine* 3: 2050312115610127. 10.1177/2050312115610127 PMC4679333 26770804

[R46] Ubink J . 2016. Access vs. justice: Customary courts and political abuse—lessons from Malawi’s Local Courts Act. *The American Journal of Comparative Law* 64: 745–84.

[R47] Ubink J, Weeks SM. 2017. Courting custom: regulating access to justice in rural South Africa and Malawi. *Law & Society Review* 51: 825–58.

[R48] United Nations . 2016. The Sustainable Development Goals Report 2016. 10.18356/3405d09f-en, accessed 2 May 2023.

[R49] Vian T . 2008. Review of corruption in the health sector: theory, methods and interventions. *Health Policy and Planning* 1: 83–94. 10.1093/heapol/czm048 18281310

[R50] Vian T . 2013. Complaints mechanisms in health organizations. *U4 Brief* 6: 1.

[R51] World Bank . 1989. Sub-Saharan Africa: from Crisis to Sustainable Growth. *A Long-Term Perspective Study*. Washington, DC: World Bank.

[R52] World Health Organization . 2000. *The World Health Report 2000*: *Health systems: improving performance*. Geneva: World Health Organization. https://reliefweb.int/report/world/world-health-report-2000-health-systems-improving-performance, accessed 2 May 2023.

