# Correction to: ‘We stay silent and keep it in our hearts’: a qualitative study of failure of complaints mechanisms in Malawi’s health system

**DOI:** 10.1093/heapol/czae057

**Published:** 2024-07-06

This is a correction to: Maryam Chilumpha, Gertrude Chatha, Eric Umar, Martin McKee, Kerry Scott, Eleanor Hutchinson, Dina Balabanova, ‘We stay silent and keep it in our hearts’: a qualitative study of failure of complaints mechanisms in Malawi’s health system, *Health Policy and Planning*, Volume 38, Issue Supplement_2, November 2023, Pages ii14–ii24, https://doi.org/10.1093/heapol/czad043

In the originally published version of the manuscript there was an lacuna in the section **Funding**. This should read additionally: ‘This work was supported by a research grant from the Health Systems Research Initiative with funding from the UK Foreign, Commonwealth & Development Office (FCDO), the Medical Research Council (MRC) and Wellcome, with support from the UK Economic and Social Research Council, (grant no. MR/T023589/1).’

The emendation has been made to the article.

