# Supplementary material for: ‘We stay silent and keep it in our hearts’: a qualitative study of failure of complaints mechanisms in Malawi’s health system
Source: Health Policy Plan. 2023 Nov 16;38(Suppl 2):ii14–24. doi: 10.1093/heapol/czad043 (PMC10666912; doi:10.1093/heapol/czad043)
Supplement: czad043_Supp [file czad043_supp.zip › suppl_data/FINAL version supplementary table.docx]

***Table 1:*** ***Potential grievance redress mechanisms for healthcare issues in Malawi at the community, health facility, district and national levels in detail***

| **COMMUNITY COMPLAINTS MECHANISMS** | | |
| --- | --- | --- |
| **Mechanism** | **Description** | **Method of Complaints Handling*** |
| Traditional leaders | - Traditional leadership is a hereditary hierarchical system within a geographical area that consists of a paramount chief, senior chief (traditional authority), chief, sub-chief, group village headman and village headman. - A paramount chief is a figure that rules over an entire tribe or ethnic group. As of 2009 there have been 7 paramount chiefs. Not all tribes have a paramount chief. - A traditional authority oversees several areas within a district. - A group village headman is a leader for several village while a village headman heads a village. - In particular traditional authorities, group village headmen and village headmen have been used as a means of handling community complaints since pre-colonial days because they are the custodians of customary law which continues to govern many aspects of life especially although not exclusively in rural areas. - These traditional leaders answer to the one that is above them. - However, traditional leaders do not have jurisdiction within cities, municipalities and townships unless given the authority by their local government councils. | Advocacy-based mechanisms:  Can refer complaints to other bodies and advocate for redressal |
| Area Development Committees (ADC)/Community Development Committees (CDC) | - ADCs and CDCs are the same in how they function. The only difference is that ADCs are found in rural areas whereas CDCs belong to urban settings. - They are multisectoral legal decentralization entities of the local government and are linked to district/city/ municipality councils. - It was formed under the guidance of a traditional authority. - It is formed every five years when a new government comes into power. - They compose of the village development committee chairpersons, representatives of public institutions, political parties, faith groups, women and children. The secretary is an employee of the council (Jones et al. 2013, MLGRD 2013). - While not all area development committees work well, interviews with officials suggests that ADC chairs do question district officials and their Members of Parliament about how funds are spent. - This places them in a good position to receive health related complaints from community members that have not been resolved at local level and ADC chairs with local government experience are better able to call the district to account but they lack capacity and power to do so (Jones et al 2013). - They answer to the Director of Planning and Development at their local council and they all report to their local traditional authority. | Advocacy-based mechanisms:  Can refer complaints to other bodies and advocate for redressal |
| Village Health Committees | - A group of 10 people from a village with half being women and the rest comprising of youth, faith representatives and those living with a disability. - It is formed under the guidance of a local health surveillance. - Solely focused on health-related issues through promoting different activities including health awareness and prevention in their villages. - They collaboratively, work hand in hand with health facilities through health surveillance assistants by moving door by door (Jones 2013). - This is also an opportunity for those health users that have complaints to lay their grievances with them as they are easily accessible in every village and are well known. | Advocacy-based mechanisms:  Can refer complaints to other bodies and advocate for redressal |
| Religious leaders | - They are respected and trusted leaders of their faith groups. - Depending on the faith group they belong to when they voice out their concerns to government they get taken seriously. - As such, at local level they are able to receive complaints from their followers then they pass them on to the right authorities. - Some also serve in community committees. | Advocacy-based mechanisms:  Can refer complaints to other bodies and advocate for redressal |
| Member of parliament | - Democratically elected officials who come into power every five years. - They are part of the legislative arm of government. - They are part of the decentralized local government system where they have the power to vote on matters being debated. - They are members of parliament who represent their constituencies and are expected to prioritize the interests of the communities they serve. They should be easily accessible by their constituents who can approach them in person and/or contact them via a call or text. | Advocacy-based mechanisms:  Can refer complaints to other bodies and advocate for redressal |
| Ward Councillor | - Democratically elected officials who assume office for five years. - They are part of the decentralized local government system. - They represent the interests and concerns of their ward at their local council. - They make sure their wards are developed with a fair distribution of resources. - They ensure that there is transparency and accountability in the running of the councils. - They also provide feedback to those they represent. - They report to the mayor who assumes office for two and half years. Who have been elected by fellow councillors. - They are required to collaborate with local MPs on developmental projects. - They also advocate for their representatives to their local MPs on the challenges that they are encountering. | Advocacy-based mechanisms:  Can refer complaints to other bodies and advocate for redressal |
| Traditional Courts/Local Courts | - They have been in existence since pre-colonial days. - A chief presides over the court who uses customary law. - In the 1970s and 1980s they were given the jurisdiction to prosecute both civil and criminal matters. However, they became corrupt as they became a tool for prosecuting political opponents. - Over time their operations were suspended and many of them have become magistrate courts. - In 2011, a law was passed in parliament to reintroduce them so that they could adjudicate minor civil and criminal cases but due to funding they are yet to be established. - Informal traditional courts exist that apply customary law but they are only able to mediate or resolve disputes. They do not have adjudication powers. | Mediating mechanisms:  can mediate disagreements |
| Malawi Police Services | Established under section 152 of the Constitution; governed by the Police Act   - Are independent and have a wide law enforcement power. - This constitutionally guaranteed independence lays a good foundation for the proper functioning of the police without interference. - They are under the ministry of homeland security. - They do mediate-resolve disputes at local level and they exercise power to fine or arrest where the matter is criminal. | Mediate-resolve |
| **HEALTH FACILITY COMPLAINTS MECHANISMS** | | |
| Suggestion Box | - Were officially introduced in 2022 in all health facilities. - They are the best option for those that want anonymity. - They are opened once a month in the presence of the Health Centre Advisory Committee, officer in charge and ombudsman. | Advocacy-based mechanisms:  Can refer complaints to other bodies and advocate for redressal |
| Hospital Ombudsman | - A collaboration between the ministry of health and population and the office of the ombudsman to mediate-resolve health user complaints - They report to the district hospital ombudsman. - A hospital ombudsman is a health surveillance assistance. - The reason for making a health surveillance assistance as a hospital ombudsman was because they are many in numbers that work with a health facility as well as the community. Due to funding, it made sense to use health surveillance assistances as hospital ombudsmen to fill the demand for complaints redressal. - They are an internal channel that is required to address complaints right there and then. - They are expected to speed up the process of redressal. - They are beginning to become a recognisable means of complaints because of media promotion. - They work hand in hand with the officer in charge and the subcommittee of the health centre advisory committee on complaints. | Employment-based mechanisms:  can suspend, transfer or fire health workers |
| Health Centre Advisory Committees | - They are a health facility committee that is a bridge between a health centre and the community. - The committee comprises of both community members who make up the majority of 15 out of 19 seats and the remaining 4 are taken up by health workers. - They discuss with the health facility on matters pertaining to the health facility and the community. - They also supervise and co-sign on the delivery of drugs and other medical supplies. - They handle health user complaints against health providers and/or the health facility that they serve. | Employment-based mechanisms:  can suspend, transfer or fire health workers |
| Health Facility Officer in Charge | - They are either a medical doctor, medical officer or a medical assistant - At primary healthcare level they report to their local DHSS. - They are in charge of a health facility. Complaints can be lodged through them. - They have a direct link to the Directorate of Health and Social Services Office and it is easy for them to take complaints there should they fail to resolve them internally. | Employment-based mechanisms:  can suspend, transfer or fire health workers |
| **DISTRICT COMPLAINTS MECHANISMS** | | |
| District Council/District Commissioner | - There are 28 district councils in Malawi - Decentralization has shifted more power from central government to district councils. - This has meant that district councils have more flexibility in decision making and how resources should be distributed. As such, problems that arise are dealt with at district level. District councils are run by the district executive committee that is headed by the district commissioner. - Local government finance committees are responsible for budgeting and managing finances at district level. - councils are under the ministry of local government and rural development. - They work towards providing communities with adequate services. - They make budget and development plans for their districts. - They also give advice to the councillors. | Employment-based mechanisms:  can suspend, transfer or fire health workers |
| City council | - There are 4 city councils. - Decentralization has shifted more power from central government to city councils. This has meant that city councils have more flexibility in decision making and how resources should be distributed. - City councils are a part of the Local Government System. As such, problems that arise are dealt with at urban level. - City councils are run by the urban executive committee that is headed by the chief executive officer. - Local government finance committees are responsible for budgeting and managing finances at urban level. - They report to the ministry of local government and rural development. - They are headed by a chief executive officer. | Employment-based mechanisms:  can suspend, transfer or fire health workers |
| Directorate of Health and Social Services (District Health Office) | - The Directorate of Health and Social Services (DHSS or commonly known as DHO) is part of the district executive committee. - It is under its local district council. - It is headed by the Director of Health and Social Services - It over sees primary healthcare facilities and the district hospital. - Blantyre DHSS is unique as it stands on its own and is not attached to any district hospital. - This is because Blantyre does not have a district hospital. - Complaints at DHSS level are handled according to the cadre that the accused belongs to. - If it is related to anyone that falls within the practice of medicine then the matter is addressed by the district medical officer who comes second after the director of health and social services. - When it relates to nurses then the district nursing officer handles that. - The district pharmacy officer handles pharmacy issues and the district environmental officer comes in when it is to do with health surveillance assistances and officers. | Employment-based mechanisms:  can suspend, transfer or fire health workers |
| District Hospital Ombudsman | - The head of hospital ombudsmen at district level. - They resolve complaints at district level when all others have failed. - Their main role is the district human resources manager. - The district health ombudsman also directly receives and handles complaints at district level. | Employment-based mechanisms:  can suspend, transfer or fire health workers |
| Judiciary (Lower Grade 1, 2, 3, 4 Magistrate Courts) | - Magistrate courts also have the power to adjudicate cases at this level. - They are part of the judiciary arm of government. - There are 29 magistrate courts across the country. | Legal mechanisms:  can charge or convict health workers |
| **NATIONAL COMPLAINTS MECHANISMS** | | |
| Ministry of Health and Population/Minister of Health and Population |  | Employment-based mechanisms:  can suspend, transfer or fire health workers |
| Ministry of Local Government and Rural Development/Minister of Local Government and Rural Development |  | Employment-based mechanisms:  can suspend, transfer or fire health workers |
| NGO/Rights-Based Organizations |  | Advocacy-based mechanisms:  Can refer complaints to other bodies and advocate for redressal |
| Drug Theft Investigation Unit | - It was established by the Ministry of Health and Population after the US government and other donors threatened to pull out funding. - It is meant to counteract drug theft in the health sector. - It consists of auditors from the ministry of health and population as well as some police officer. They also collaborate with the fiscal police and the pharmacy, medicine and poisons board. - It was mainly operational between 2016 and 2017. | Advocacy-based mechanisms:  Can refer complaints to other bodies and advocate for redressal |
| Quality Management Directorate (To improve healthcare quality delivery) | - It was established in 2016 in the Ministry of Health and Population to provide strategic leadership and coordinate quality management and improve initiatives in the health sector. - It supports the hospital ombudsman through training and coordination. - It also uses the complaints from hospital ombudsmen to improve the quality of healthcare delivery as well as policy revision. | Advocacy-based mechanisms:  Can refer complaints to other bodies and advocate for redressal |
| Anti-Corruption Bureau | - It was established under section 4 of the Corrupt Practices Act. - It is under the ministry of justice. - it has offices in all four cities of Malawi and its headquarters are in Lilongwe. - The director is appointed by the president and is subject to confirmation by the Public Appointments Committee of parliament. - It is an institution that handles multi-sectoral corruption. - It is legally mandated to act without interference of any person or authority. A good foundation towards ensuring the independence of the ACB. - A matter upon investigation is either taken to court or dealt with officials from the institution or person (s) in question. | Employment-based mechanisms:  can suspend, transfer or fire health workers |
| Malawi Police Services |  | Legal mechanisms:  can charge or convict health workers |
| Ombudsman | - It was established under section 122 of the Constitution. - The office has a wide range of powers and functions to remedy any injustices suffered by any person. - The Constitution empowers the ombudsman to direct administrative action**,** to order the appropriate authorities to provide for redress of future grievances and to refer matters for prosecution. | Employment-based mechanisms:  can suspend, transfer or fire health workers |
| National Local Government Finance Committee | - It was established under section 149 of the Constitution and the Local Government Act (1998) further guarantees its powers and functions. - Its office is in the capital Lilongwe. - It is the overseer of all Local Government Finance Committees. - The Local Government Act gives the LGFC an oversight role in the finance management of district councils and consequently, the entire local government area. - Though the Local Government Act gives LGFC an oversight role in finance management, councils have the discretion to decide who can be part of this crucial entity and on what terms such membership will be. - Complaints can only be made through the National Local Government Finance Committee in the capital city Lilongwe. - The other methods are to give them a call or to send a complaint via postal mail. | Advocacy-based mechanisms:  Can refer complaints to other bodies and advocate for redressal |
| Office of the Auditor General | - It was established under section 184 of the Constitution; governed by the Public Audit Act. - Appointment and Removal is done by the president of the Republic of Malawi. - It reports to the National Assembly. - It is meant to enjoy Independence and non-interference | Advocacy-based mechanisms:  Can refer complaints to other bodies and advocate for redressal |
| Judiciary (Supreme Court of Appeal, High Court, Chief Resident Magistrates, who head the magistracy in each of the judicial regions; Principal Resident Magistrates; Senior Resident Magistrates; Resident Magistrates) | - An arm of government. - Headed by the Chief Justice. - It has three sections namely the Supreme Court of Appeal which is the highest, followed by the High Court which has unlimited original jurisdiction to hear and determine any civil or criminal under any law. - Both the High courts and these Magistrate courts are found in all 4 cities of Malawi, Blantyre, Lilongwe, Mzuzu and Zomba. - The Supreme Court of Appeal presides in Blantyre. - These courts fall under the Judicial Service Commission. | Legal mechanisms:  can charge or convict health workers |
| Medical Council of Malawi | - It was established under The Medical Practitioners and Dentists Act No 17 of 1987. - It is the only entity in the country that registers medical practitioners, paramedical and allied health providers. - The President appoints members to the Council and the Registrar heads the secretariat. - The Council works in committees i.e., the disciplinary committee. - They meet quarterly a year. - Their main source of funding comes from government. - The disciplinary committee addresses complaints from health users and guardians against medical practitioners and health facilities. - They can legally adjudicate as they have the same status as a magistrate court. - In part they also use health user complaints as a means of promoting high quality medical practice. | Legal mechanisms:  can charge or convict health workers |
| Nurses and Midwives Council of Malawi | - The sole regulatory body for nursing and midwifery education, training, practice and professional conduct of nurses and midwives which was established under the laws of Malawi through the Nurses and Midwives Act. - It is mandated to protect the public from being ill-treated by nurses and midwives. - It function in committees i.e., one of them being a disciplinary committee. - It convenes quarterly a year. - It has adjudication powers like a magistrate court and if a health user or nurse is not satisfied with the verdict, they are free to appeal with the high court. Some of the ways in which they receive complaints is as follows: - They get referrals from the Ministry of Health, Medical Council of Malawi, supervisors from health facilities - Complainants are also expected to call via their toll-free line and they do advertise about this line through posters which they paste in all public health facilities but it is suspected that health providers at these facilities take them down. - Formal complaints are lodged through a postal letter or an email. - These complaints are presented at the disciplinary committee to determine if it is worth investigating or not. | Legal mechanisms:  can charge or convict health workers |
| Pharmacy and medicines Regulatory Authority (PMRA) | - it was established under the Pharmacy and Medicines Regulatory Authority (PMRA) Act of 2019. - It succeeds the Pharmacy, Medicines and Poisons Board which was established under the Pharmacy, Medicines and Poisons Act of 1988. - It is the sole regulator of the, education, training and practice of pharmacy. It is also involved in the registration and disciplining of pharmacists, pharmacy technologists and pharmacy assistants and medical representatives, their training; among many of other areas of pharmacy in Malawi. | Legal mechanisms:  can charge or convict health workers |
| Parliamentary Committee on Health |  | Legal mechanisms:  can charge or convict health workers |
